# Potential influencing factors on the outcome in incisional hernia repair: a registry-based multivariable analysis of 22,895 patients

**DOI:** 10.1007/s10029-020-02184-9

**Published:** 2020-04-10

**Authors:** F. Köckerling, H. Hoffmann, D. Adolf, W. Reinpold, P. Kirchhoff, F. Mayer, D. Weyhe, B. Lammers, K. Emmanuel

**Affiliations:** 1 Department of Surgery and Center for Minimally Invasive Surgery, Academic Teaching Hospital of Charité Medical School, Vivantes Hospital, Neue Bergstrasse 6, 13585 Berlin, Germany; 2 Two Surgeons-Center for Hernia Surgery and Proctology, St. Johanns-Vorstadt 44, 4056 Basel, Switzerland; 3 StatConsult GmbH, Halberstädter Strasse 40 a, 39112 Magdeburg, Germany; 4 Department of Surgery, Wilhelmsburger Hospital Gross Sand, Academic Teaching Hospital of University Hamburg, Gross Sand 3, 21107 Hamburg, Germany; 5 Department of Surgery, Paracelsus Medical University Salzburg, Müllner Hauptstrasse 48, 5020 Salzburg, Austria; 6 Department of General and Visceral Surgery, Pius Hospital, University Hospital of Visceral Surgery, Georgstrasse 12, 26121 Oldenburg, Germany; 7 Department of Surgery I–Section Coloproctology and Hernia Surgery, Lukas Hospital, Preussenstrasse 84, 41464 Neuss, Germany

**Keywords:** Incisional hernia, Outcome, EHS-classification, Laparoscopic IPOM, Sublay, Chronic pain

## Abstract

**Introduction:**

Due to the paucity of randomized controlled trials, meta-analyses of incisional hernia repair can hardly give any insights into the influence factors on the various outcome criteria. Therefore, a multivariable analysis of data from the Herniamed Registry was undertaken with the aim to define potential influencing factors for the outcome.

**Methods:**

Multivariable analysis of the data available for 22,895 patients with primary elective incisional hernia repair was performed to assess the confirmatory predefined potential influence factors and their association with the perioperative and 1-year follow-up outcomes. A model validation procedure was implemented using a bootstrap algorithm in order to account for the robustness of results.

**Results:**

Higher European Hernia Society (EHS) width classification, open procedure, female gender, and preoperative pain have a highly significant association with an unfavorable outcome in incisional hernia repair. Larger defect width and open operation have a highly significantly unfavorable relation to the postoperative surgical complications, general complications, and the complication-related reoperations, while female gender and preoperative pain have a highly significantly unfavorable association with the rates of pain at rest, pain on exertion, and chronic pain requiring treatment at 1-year follow-up. The recurrence rate is significantly unfavorably influenced by higher EHS width classification, higher BMI, and lateral EHS classification.

**Conclusion:**

Higher EHS width classification, open procedure, female gender, higher BMI, and lateral EHS classification, as well as preoperative pain are the most important unfavorable influencing factors associated with a worse outcome in incisional hernia repair.

## Introduction

Compared with primary ventral hernia repair, incisional ventral hernia repair has a significantly poorer perioperative and long-term outcomes [1–6]. Therefore, data on primary and incisional ventral hernias should not be jointly evaluated in studies [1–6]. Hence, meta-analysis findings that make no distinction between primary and incisional ventral hernia repair should be interpreted with caution [7]. The two meta-analyses focusing exclusively on incisional hernia were able to evaluate only a maximum of six randomized controlled trials with a total of 751 patients [8–11]. That small sample size with a relatively large number of factors potentially influencing the outcome is hardly suitable for reliable identification of the relevance of the various influence factors on the outcome in incisional hernia repair. The meta-analysis was only able to demonstrate that the wound complication rate in laparoscopic intraperitoneal onlay mesh (IPOM) was lower than in the open procedures [8–11]. There was no significant difference in the recurrence rates [8–11].

Registry and database analyses reported in the literature make several references to factors that have an unfavorable influence on the outcome following incisional hernia repair [12–16]. These unfavorable factors, include age, gender, risk factors, open surgical procedures, defect width, body mass index (BMI) ≥ 30 kg/m^2^), and smoking [12–16]. Recently, an analysis of the data of 2191 patients from the French Hernia Registry “Club Hernie” showed that larger defect widths, as classified by the European Hernia Society (EHS) [17], had an unfavorable impact on the postoperative complication rate [15].

This present analysis of data from the Herniamed Hernia Registry [18, 19] aims to assess potential influencing factors associated with outcome in incisional hernia repair. In particular, it seeks to evaluate the importance of the European Hernia Society width classification [17] as an unfavorable factor for the outcome in incisional hernia repair. To do so, the impact of confirmatory chosen, potential influencing factors on several outcome parameters was assessed, accounting for notable odds ratios and robust results.

## Materials and methods

“The Herniamed quality assurance study is a multicenter internet-based hernia registry with voluntary participating institutions which incorporate prospective data of patients who have undergone routine hernia surgery” [20, 21].

“These data are obtained from 712 voluntarily participating hospitals and surgeons in Germany, Austria, and Switzerland” [20, 21]. All patients gave informed consent agreeing to participate [20, 21]. “As part of the informed consent declaration, information provided to patients regarding participation in the Herniamed Registry included the request that the hospital or medical practice providing treatment would like to be informed about any problem occurring after the operation and that patients have the opportunity to attend clinical examination” [20, 21].

“At 1-year follow-up, postoperative complications are once again reviewed when the general practitioner and patient are asked to report any occurrences, pain at rest, pain on exertion, and chronic pain requiring treatment” [20, 21]. “If recurrence or chronic pain is reported by the patient or the general practitioner the patients can be requested to present themselves for clinical or radiological examination” [20, 21]. A publication by Baucom et al. [22] has provided impressive evidence of the role of patient-reported outcomes for both recurrence and chronic pain following incisional hernia repair.

In the current analysis, prospective data of patients who underwent primary elective incisional hernia repair with the laparoscopic intraperitoneal onlay mesh (IPOM) technique or open suture, sublay, onlay, or IPOM approach were evaluated to assess all confirmatory pre-defined potential influencing factors on the perioperative and 1-year follow-up outcomes. Here, the focus in particular was to assess the role of EHS width classification W1 (< 4 cm), W2 (≥ 4 cm–10 cm), W3 (> 10 cm) on the outcome [16]. Further variables to be assessed were age in years, BMI in kg/m^2^, gender, ASA score, surgical technique, preoperative pain (yes, no, unknown), drainage (yes, no), EHS classification (medial, lateral, combined), presence of risk factors (yes, no), and postoperative complications (yes, no) on analysis of pain at follow-up.

Risk factors were deemed to apply if at least one of the following risk factors was present: COPD, diabetes mellitus, aortic aneurysm, immunosuppression, corticoids, smoking, coagulopathy, antiplatelet medication not adequately discontinued, or coumarin derivatives with Quick/INR not in normal range.

**Fig. 1:**
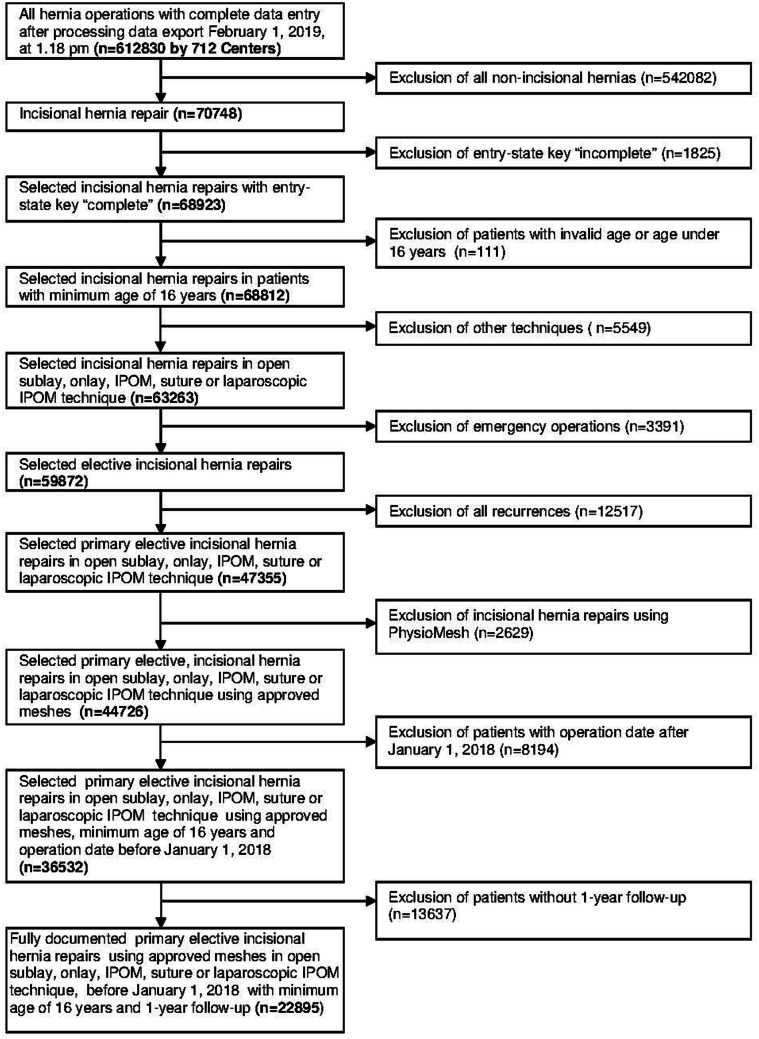
Flowchart pf patient inclusion

The main inclusion criteria for the analysis population were minimum valid age of 16 years, primary elective incisional hernia repair using the laparoscopic IPOM or open suture, sublay, onlay, IPOM technique, no use of a Physiomesh [20], and availability of data at 1-year follow-up (Fig. 1). 22,895 patients fulfilled these inclusion criteria (Fig. 1).

Physiomesh was excluded from this analysis because of the voluntary market withdrawal [20]. Recurrent incisional hernias were also excluded for homogeneity of the patient population.

In total, 22,895 patients were selected between September 1, 2009 and January 1, 2018. Of these patients, 6361 (27.8%) had undergone laparoscopic IPOM, 2662 (11.6%) open suture, 9378 (41.0%) open sublay, 3196 (13.9%) open IPOM, and 1298 (5.7%) open onlay procedure.

All analyses were performed with the software SAS 9.4 (SAS Institute Inc. Cary, NC, USA) and deliberately reviewed to the full level of significance. Each *p* value ≤ 0.05 thus represents a statistically significant result. Categorical variables are given as absolute and relative frequencies. For continuous data, mean and standard deviation or range of dispersion for log-transformed data, respectively, are given.

For unadjusted analyses of EHS width classification, the Chi-square test was used for categorical variables and an analysis of variance (ANOVA) was used for continuous variables. Analyses of non-normal distributed data (operating time and mesh size) were done on log-transformed values.

The potential influence of EHS width classification on the outcome parameters (intraoperative, postoperative and general complications, complication-related reoperations, as well as recurrence, pain at rest, pain on exertion, and pain requiring treatment at 1-year follow-up) adjusted for pre-defined confounding patient- and operation-related variables was analyzed via multivariable binary logistic models. Estimates for odds ratio (OR) and the corresponding 95% confidence interval are given. For independent variables with more than two categories, all pairwise odds ratios are provided. For the continuous variable “age” the 10-year odds ratio, and for the variable “BMI” a five-point odds ratio, is given.

All available data were included in the models. Detailed results of outcome variables presented in this paper refer to the corresponding estimates in those single models. The robustness of the results in terms of stable odds ratio estimates was assessed using a bootstrap algorithm per model with 1000 bootstrap samples each.

**Fig. 2:**
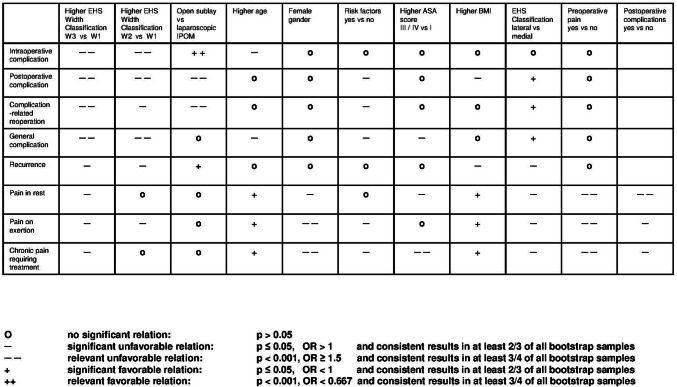
Scheme of relationship between outcomes and potential influencing factors including information from bootstrap algorithm (not all pairwise comparisons shown)

Since significance can also be reached for very small effects in such a huge registry study, in order to facilitate evaluation of the relevance of individual influence variables on the various outcome criteria, and to account for robustness of results, the following definition of influence strength is applied in the summary presentation of results (Fig. 2):

- An odds ratio of ≥ 1.5 with a corresponding *p* value of < 0.001 and consistent results in at least 3/4 of all bootstrap samples are defined as highly significantly unfavorable relation.
- An odds ratio of < 0.667 with a corresponding *p* value of < 0.001 and consistent results in at least 3/4 of all bootstrap samples refer to a highly significantly favorable relation.
- A significant effect (*p* ≤ 0.05) with an odds ratio of > 1 and consistent or even strengthened results in at least 2/3 of all bootstrap samples indicate a significantly unfavorable relation
- A significant effect (*p* ≤ 0.05) with an odds ratio of < 1 and consistent or even strengthened results in at least 2/3 of all bootstrap samples define a significantly favorable relation.

## Results

### Unadjusted analysis

This section investigated deviations in the frequency distribution of the influence and outcome variables in relation to EHS width classification unadjusted for other potential influencing factors. The frequency distribution for EHS width classification revealed for W1 (< 4 cm) *n* = 8615 cases (37.6%), for W2 (≥ 4 cm–10 cm) *n* = 10,519 (45.9%) cases, and for W3 (> 10 cm) *n* = 3761 (16.4%) cases.

Patients with larger defects had a significantly higher age (Table 1). Likewise, the BMI (kg/m^2^) was significantly higher for larger defects. The mean operating time was notably longer for larger defects. As expected, the meshes used were on average significantly larger for higher EHS classification.

**Table 1:** Presentation of ranges and of unadjusted analysis results for homogeneity between width classification and age, BMI, operating time, and mesh size

		EHS width classification			*p*
		W1 (< 4 cm)	W2 (≥ 4–10 cm)	W3 (> 10 cm)	
Age [years]	*N*/mean ± SD	8615/60.4 ± 14.6	10,519/64.4 ± 12.5	3761/64.9 ± 11.9	< 0.001
BMI [kg/m^2^]	*N*/mean ± SD	8587/28.4 ± 5.6	10,474/29.5 ± 5.8	3747/29.9 ± 5.9	< 0.001
OP-time [min]^a^	*N*/mean [range]	7978/50.4 [48.7; 52.1]	10,492/80.5 [78.9; 82.2]	3759/110.8 [109.2; 112.3]	< 0.001
Mesh size [cm^2^]^a^	*N*/mean [range]	6274/118.4 [116.0; 120.9]	9953/270.1 [268.2; 272.1]	3643/487.2 [485.5; 488.8]	< 0.001

^a^Logarithmic transformation: illustration of the back-transformed mean values and ranges (mean value ± SD)

Although the proportion of male patients with increasing EHS width classification rose, the corresponding proportion of women declined (Table 2). Significant differences were also seen in the distribution of the operative techniques used with regard to EHS width classification. Likewise, a greater proportion of patients with higher width classification also showed higher ASA scores. The proportion of lateral EHS classifications declined in line with rising EHS width classification, whereas the proportion of combined EHS classifications increased significantly. The proportion of patients with preoperative pain declined significantly in line with increasing defect width. Patients with larger defect widths also had significantly more risk factors. Similarly, for increasing defect widths, surgeons used drains significantly more often.

**Table 2:** Presentation of descriptive statistics and of unadjusted analysis results for homogeneity between width classification and categorical influencing variables

	EHS width classification						*p*
	W1 (< 4 cm)		W2 (≥ 4–10 cm)		W3 (> 10 cm)		
	*n*	%	*n*	%	*n*	%	
Gender							
Male	4132	47.96	5245	49.86	2038	54.19	< 0.001
Female	4483	52.04	5274	50.14	1723	45.81	
Procedure							
Laparoscopic—IPOM	2469	28.66	3099	29.46	793	21.08	< 0.001
Open—suture	2223	25.80	377	3.58	62	1.65	
Open—IPOM	1219	14.15	1283	12.20	694	18.45	
Open—onlay	440	5.11	649	6.17	209	5.56	
Open—sublay	2264	26.28	5111	48.59	2003	53.26	
ASA score							
I	1460	16.95	901	8.57	242	6.43	< 0.001
II	5047	58.58	5952	56.58	1971	52.41	
III/IV	2108	24.47	3666	34.85	1548	41.16	
EHS classification							
Combined	495	5.75	846	8.04	537	14.28	< 0.001
Lateral	1716	19.92	1823	17.33	428	11.38	
Medial	6404	74.34	7850	74.63	2796	74.34	
Preoperative pain							
Yes	5178	60.10	5842	55.54	2068	54.99	< 0.001
No	2741	31.82	3745	35.60	1373	36.51	
Unknown	696	8.08	932	8.86	320	8.51	
Drainage							
Yes	2893	33.58	6748	64.15	2843	75.59	< 0.001
No	5722	66.42	3771	35.85	918	24.41	
Risk factors							
Total							
Yes	3091	35.88	4497	42.75	1794	47.70	< 0.001
No	5524	64.12	6022	57.25	1967	52.30	
COPD							
Yes	755	8.76	1134	10.78	457	12.15	< 0.001
No	7860	91.24	9385	89.22	3304	87.85	
Diabetes							
Yes	884	10.26	1482	14.09	626	16.64	< 0.001
No	7731	89.74	9037	85.91	3135	83.36	
Aortic aneurysm							
Yes	67	0.78	196	1.86	107	2.84	< 0.001
No	8548	99.22	10,323	98.14	3654	97.16	
Immunosuppression							
Yes	109	1.27	202	1.92	95	2.53	< 0.001
No	8506	98.73	10,317	98.08	3666	97.47	
Corticoid							
Yes	124	1.44	179	1.70	86	2.29	0.004
No	8491	98.56	10,340	98.30	3675	97.71	
Smoking							
Yes	1007	11.69	1225	11.65	515	13.69	0.002
No	7608	88.31	9294	88.35	3246	86.31	
Coagulopathy							
Yes	137	1.59	243	2.31	95	2.53	< 0.001
No	8478	98.41	10,276	97.69	3666	97.47	
ASS/plavix antiplatelet medication							
Yes	841	9.76	1353	12.86	535	14.22	< 0.001
No	7774	90.24	9166	87.14	3226	85.78	
Anticoagulation therapy							
Yes	248	2.88	341	3.24	123	3.27	0.293
No	8367	97.12	10,178	96.76	3638	96.73	

Unadjusted analysis of the relationship between width classification and the intra- and postoperative surgical complications, general complications and complication-related reoperations, recurrences, as well as pain at rest, and on exertion and chronic pain requiring treatment at 1-year follow-up is presented in detail in Table 3. A significant relationship was identified between width classification and all outcome variables unadjusted for other potential influences. For all outcome parameters, the corresponding rate rose to a relevant degree in line with increasing width classification.

**Table 3:** Presentation of descriptive statistics and unadjusted analysis results for homogeneity between width classification and outcome variables

	EHS width classification						*p*
	W1 (< 4 cm)		W2 (≥ 4–10 cm)		W3 (> 10 cm)		
	*n*	%	*N*	%	*n*	%	
Intraoperative complication							
Yes	87	1.01	221	2.10	93	2.47	< 0.001
No	8528	98.99	10,298	97.90	3668	97.53	
Postoperative complication							
Yes	353	4.10	863	8.20	516	13.72	< 0.001
No	8262	95.90	9656	91.80	3245	86.28	
General complication							
Yes	161	1.87	398	3.78	232	6.17	< 0.001
No	8454	98.13	10,121	96.22	3529	93.83	
Complication-related reoperation							
Yes	148	1.72	381	3.62	222	5.90	< 0.001
No	8467	98.28	10,138	96.38	3539	94.10	
Recurrence on 1-year-follow-up							
Yes	377	4.38	528	5.02	206	5.48	0.018
No	8238	95.62	9991	94.98	3555	94.52	
Pain on exertion on 1-year-follow-up							
Yes	1425	16.54	1948	18.52	763	20.29	< 0.001
No	7190	83.46	8571	81.48	2998	79.71	
Pain in rest on 1-year-follow-up							
Yes	798	9.26	1041	9.90	418	11.11	0.006
No	7817	90.74	9478	90.10	3343	88.89	
Pain requiring treatment on 1-year-follow-up							
Yes	591	6.86	806	7.66	320	8.51	0.004
No	8024	93.14	9713	92.34	3441	91.49	

### Multivariable analysis

#### Intraoperative complications

The risk of intraoperative complications (model fit: *p* < 0.001) was significantly related with the surgical technique, width classification, and the use of a drain (in each case *p* < 0.001), as well as age (*p* = 0.012) and gender (*p* = 0.024) (Table 4, Fig. 2). The open techniques were associated with a lower intraoperative complication risk. Furthermore, the use of drains and higher EHS width classifications were associated with higher intraoperative complication risk. Likewise, in older patients, there was a higher risk of intraoperative complications. Those estimated effects for EHS width classification correspond to differences of, e.g., 21 intraoperative complications for every 1000 operations with W3 as compared to nine intraoperative complications for every 1000 operations with W1 width classification.

**Table 4:** Multivariable analysis results for intraoperative complications, including odds ratio estimates with corresponding 95% confidence intervals

Parameter	*p* value	Category	*p* value (pairwise)	OR estimate	95% CI	
Procedure	< 0.001	Open—sublay vs laparoscopic—IPOM	< 0.001	0.303	0.226	0.405
		Open—suture vs open—sublay	< 0.001	3.169	2.206	4.552
		Open—IPOM vs laparoscopic—IPOM	< 0.001	0.483	0.343	0.680
		Open—onlay vs laparoscopic—IPOM	< 0.001	0.430	0.269	0.686
		Open—suture vs open—IPOM	0.001	1.987	1.319	2.992
		Open—suture vs open—onlay	0.002	2.232	1.339	3.723
		Open—IPOM vs open—sublay	0.004	1.595	1.157	2.198
		Open—onlay vs open—sublay	0.120	1.420	0.913	2.207
		Open—onlay vs open—IPOM	0.640	0.890	0.546	1.450
		Open—suture vs laparoscopic—IPOM	0.822	0.959	0.669	1.376
Drainage	< 0.001	Yes vs no		2.657	2.035	3.468
EHS width classification	< 0.001	W3 (> 10 cm) vs W1 (< 4 cm)	< 0.001	2.399	1.730	3.326
		W2 (≥ 4–10 cm) vs W1 (< 4 cm)	< 0.001	2.036	1.548	2.678
		W3 (> 10 cm) vs W2 (≥ 4–10 cm)	0.198	1.178	0.918	1.512
Age [10-years-OR]	0.012			1.117	1.024	1.218
Gender	0.024	Female vs male		1.266	1.032	1.553
EHS classification	0.272	Lateral vs medial	0.108	0.790	0.592	1.053
		Lateral vs combined	0.418	0.840	0.550	1.282
		Combined vs medial	0.730	0.941	0.664	1.333
ASA score	0.383	II vs I	0.183	0.787	0.554	1.120
		III/IV vs I	0.386	0.841	0.569	1.244
		III/IV vs II	0.570	1.068	0.851	1.341
BMI [5-points-OR]	0.455			0.966	0.883	1.057
Preoperative pain	0.818	Unknown vs no	0.527	1.124	0.782	1.616
		Yes vs unknown	0.595	0.910	0.644	1.287
		Yes vs no	0.834	1.024	0.823	1.274
Risk factors	0.840	Yes vs no		0.978	0.791	1.211

#### Postoperative complications

The analysis results for postoperative complications are presented in Table 5 (model fit: *p* < 0.001). The onset of postoperative complications was highly significantly associated with width classification, operative technique, BMI, presence of risk factors, use of a drain, and EHS classification (in each case *p* < 0.001) and significantly with ASA score (*p* = 0.002), and age (*p* = 0.041) (Fig. 2). The wider the hernia, the higher the risk of postoperative complications, which results in, e.g., 99 postoperative complications for every 1,000 operations with W3 as compared to 42 for every 1000 W1 hernias. With regards to the operative technique, a reduction in the overall postoperative complication risk was achieved by using, in particular, the laparoscopic IPOM procedure. By comparison, the open procedures exhibited significantly higher—mostly twofold—risks. Higher BMI was associated with an increase in the postoperative complication rate. In contrast, lateral EHS classification, in particular in comparison with medial EHS classification, reduced the complication risk.

**Table 5:** Multivariable analysis results for postoperative complications, including odds ratio estimates with corresponding 95% confidence intervals

Parameter	*p* value	Category	*p* value (pairwise)	OR estimate	95% CI	
EHS width classification	< 0.001	W3 (> 10 cm) vs W1 (< 4 cm)	< 0.001	2.500	2.135	2.927
		W3 (> 10 cm) vs W2 (≥ 4–10 cm)	< 0.001	1.555	1.380	1.752
		W2 (≥ 4–10 cm) vs W1 (< 4 cm)	< 0.001	1.608	1.397	1.850
Procedure	< 0.001	Open—sublay vs laparoscopic—IPOM	< 0.001	2.291	1.917	2.738
		Open—onlay vs laparoscopic—IPOM	< .001	2.449	1.914	3.133
		Open—IPOM vs laparoscopic—IPOM	< 0.001	1.977	1.622	2.410
		Open—suture vs laparoscopic—IPOM	< 0.001	1.739	1.362	2.220
		Open—suture vs open—sublay	0.013	0.759	0.611	0.943
		Open—suture vs open—onlay	0.014	0.710	0.540	0.934
		Open—IPOM vs open—sublay	0.051	0.863	0.744	1.001
		Open—onlay vs open—IPOM	0.063	1.239	0.988	1.552
		Open—suture vs open—IPOM	0.289	0.880	0.694	1.115
		Open—onlay vs open—sublay	0.504	1.069	0.879	1.300
BMI [5-points-OR]	< 0.001			1.148	1.100	1.198
Drainage	< 0.001	Yes vs no		1.436	1.244	1.657
Risk factors	< 0.001	Yes vs no		1.293	1.163	1.437
EHS classification	< 0.001	Lateral vs medial	< 0.001	0.727	0.625	0.844
		Lateral vs combined	0.066	0.812	0.650	1.014
		Combined vs medial	0.232	0.895	0.746	1.074
ASA score	0.002	III/IV vs II	< 0.001	1.216	1.086	1.360
		III/IV vs I	0.030	1.273	1.023	1.583
		II vs I	0.656	1.047	0.856	1.280
Age [10-years-OR]	0.041			1.047	1.002	1.094
Gender	0.248	Female vs male		0.941	0.850	1.043
Preoperative pain	0.441	Yes vs no	0.204	1.074	0.962	1.198
		Yes vs unknown	0.662	1.042	0.866	1.255
		Unknown vs no	0.764	1.030	0.849	1.250

### Complication-related reoperations

The analysis results for the complication-related reoperations (model fit; *p* < 0.001) (Table 6, Fig. 2) showed that the risk of complication-related reoperation was significantly associated with hernia width, use of a drain, presence of risk factors, surgical technique (in each case *p* < 0.001), EHS classification (*p* = 0.003), as well as ASA score, and BMI (*p* = 0.019). The complication-related reoperation risk—like the postoperative complication rates above—was notably related to higher width classification, resulting in, e.g., addition of 21 cases for every 1000 hernias with W3 (40/1000) as compared to W1 (19/1000). The use of drains, as well as the presence of at least one risk factor was also associated with a higher complication-related reoperation risk, whereas on comparing the operative techniques, in particular the use of a laparoscopic IPOM procedure reduced the risk. The latter also applied for lateral EHS classification, whereas higher ASA score and higher BMI were associated with a higher risk.

**Table 6:** Multivariable analysis results for complication-related reoperations, including odds ratio estimates with corresponding 95% confidence intervals

Parameter	*p* value	Category	*p* value (pairwise)	OR estimate	95% KI	
EHS width classification	< 0.001	W3 (> 10 cm) vs W1 (< 4 cm)	< 0.001	2.219	1.756	2.804
		W3 (> 10 cm) vs W2 (≥ 4–10 cm)	< 0.001	1.438	1.209	1.711
		W2 (≥ 4–10 cm) vs W1 (< 4 cm)	< 0.001	1.543	1.251	1.903
Drainage	< 0.001	Yes vs no		1.851	1.480	2.315
Risk factors	< 0.001	Yes vs no		1.439	1.231	1.683
Procedure	< 0.001	Open—sublay vs laparoscopic—IPOM	< 0.001	1.927	1.475	2.516
		Open—onlay vs laparoscopic—IPOM	< 0.001	1.973	1.367	2.846
		Open—IPOM vs laparoscopic—IPOM	0.002	1.594	1.180	2.155
		Open—suture vs open—sublay	0.037	0.698	0.497	0.979
		Open—suture vs open—onlay	0.073	0.681	0.448	1.036
		Open—IPOM vs open—sublay	0.094	0.827	0.663	1.033
		Open—suture vs laparoscopic—IPOM	0.127	1.344	0.919	1.966
		Open—onlay vs open—IPOM	0.214	1.237	0.884	1.731
		Open—suture vs open—IPOM	0.367	0.843	0.582	1.222
		Open—onlay vs open—Sublay	0.873	1.024	0.768	1.365
EHS classification	0.003	Lateral vs medial	< 0.001	0.675	0.535	0.851
		Lateral vs combined	0.012	0.659	0.476	0.911
		Combined vs medial	0.855	1.024	0.794	1.321
ASA score	0.016	III/IV vs II	0.004	1.273	1.079	1.502
		III/IV vs I	0.171	1.249	0.908	1.719
		II vs I	0.901	0.981	0.730	1.319
BMI [5-points-OR]	0.019			1.079	1.013	1.149
Gender	0.254	Female vs male		0.916	0.788	1.065
Preoperative pain	0.313	Yes vs no	0.189	1.115	0.948	1.312
		Unknown vs no	0.227	1.186	0.899	1.563
		Yes vs unknown	0.648	0.941	0.723	1.224
Age [10-years-OR]	0.836			0.993	0.932	1.059

### General complications

The general complications (model fit: *p* < 0.001) were significantly related to width classification, presence of risk factors, age, use of a drain, and ASA score (in each case *p* < 0.001), as well as EHS classification (*p* = 0.003) (Table 7, Fig. 2). As in the case of the postoperative complications—also in the effect size for somewhat lower prevalence—wider hernias increased the risk of general complications between 48 and 140%. The latter revealed 25 more cases with general complications for every 1,000 hernias with W3 (44/1000) as compared to W1 width. Independently of the above, risk factors, higher age and higher ASA score, and the use of a drain were associated with higher general complication risk. Conversely, lateral EHS classification showed a reduced complication risk.

**Table 7:** Multivariable analysis results for general complications, including odds ratio estimates with corresponding 95% confidence intervals

Parameter	*p* value	Category	*p* value (pairwise)	OR estimate	95% CI	
EHS width classification	< 0.001	W3 (> 10 cm) vs W1 (< 4 cm)	< 0.001	2.400	1.914	3.011
		W2 (≥ 4–10 cm) vs W1 (< 4 cm)	< 0.001	1.621	1.325	1.983
		W3 (> 10 cm) vs W2 (≥ 4–10 cm)	< 0.001	1.481	1.249	1.756
Risk factors	< 0.001	Yes vs no		1.500	1.288	1.747
Age [10-years-OR]	< 0.001			1.169	1.095	1.248
ASA score	< 0.001	III/IV vs II	< 0.001	1.431	1.221	1.677
		III/IV vs I	0.016	1.508	1.078	2.110
		II vs I	0.747	1.054	0.767	1.449
Drainage	< 0.001	Yes vs no		1.516	1.238	1.855
EHS classification	0.003	Lateral vs medial	< 0.001	0.679	0.544	0.849
		Lateral vs combined	0.012	0.670	0.490	0.914
		Combined vs medial	0.907	1.015	0.794	1.297
Procedure	0.175	Open—IPOM vs laparoscopic—IPOM	0.023	1.344	1.042	1.733
		Open—IPOM vs open—sublay	0.049	1.229	1.001	1.508
		Open—Onlay vs Open—IPOM	0.151	0.776	0.550	1.097
		Open—suture vs open—IPOM	0.287	0.836	0.602	1.162
		Open—sublay vs laparoscopic—IPOM	0.450	1.094	0.867	1.380
		Open—suture vs laparoscopic—IPOM	0.486	1.124	0.809	1.560
		Open—suture vs open—onlay	0.724	1.077	0.714	1.625
		Open—onlay vs open—sublay	0.770	0.954	0.696	1.307
		Open—onlay vs laparoscopic—IPOM	0.817	1.044	0.727	1.498
		Open—suture vs open—sublay	0.863	1.027	0.755	1.397
Gender	0.239	Female vs male		1.092	0.943	1.264
Preoperative pain	0.394	Yes vs no	0.198	1.109	0.947	1.299
		Unknown vs no	0.371	1.132	0.863	1.485
		Yes vs unknown	0.878	0.980	0.756	1.270
BMI [5-points-OR]	0.894			0.996	0.933	1.062

### Recurrence

The multivariable analysis results for analysis of recurrence at 1-year follow-up are given in Table 8 (model fit: *p* < 0.001). The recurrence was strongly associated with surgical technique, EHS width classification, EHS classification, and BMI (in each case *p* < 0.001) (Fig. 2). The recurrence rate was increased, in particular, by the use of the open procedure with direct suture. Open sublay had a lower recurrence risk when compared with laparoscopic IPOM. Furthermore, larger defects, lateral EHS in comparison with medial and higher BMI were associated with higher recurrence risk. Here, the difference in recurrences is about 20 cases for every 1000 hernias with W3 (57/1000) as compared to W1 (37/1000).

**Table 8:** Multivariable analysis results for recurrence at 1-year follow-up, including odds ratio estimates with corresponding 95% confidence intervals

Parameter	*p* value	Category	*p* value (pairwise)	OR estimate	95% CI	
Procedure	< 0.001	Open—suture vs open—sublay	< 0.001	2.928	2.389	3.590
		Open—suture vs laparoscopic—IPOM	< 0.001	2.316	1.884	2.846
		Open—suture vs open—IPOM	< 0.001	2.005	1.603	2.509
		Open—suture vs open—onlay	< 0.001	2.006	1.500	2.682
		Open—IPOM vs open—sublay	< 0.001	1.460	1.207	1.766
		Open—onlay vs open—sublay	0.004	1.460	1.127	1.890
		Open—sublay vs laparoscopic—IPOM	0.016	0.791	0.653	0.958
		Open—IPOM vs laparoscopic—IPOM	0.177	1.155	0.937	1.423
		Open—onlay vs laparoscopic—IPOM	0.324	1.154	0.868	1.535
		Open—onlay vs open—IPOM	0.998	1.000	0.752	1.329
EHS width classification	< 0.001	W2 (≥ 4–10 cm) vs W1 (< 4 cm)	< 0.001	1.417	1.213	1.656
		W3 (> 10 cm) vs W1 (< 4 cm)	< 0.001	1.548	1.270	1.886
		W3 (> 10 cm) vs W2 (≥ 4–10 cm)	0.305	1.092	0.923	1.293
EHS classification	< 0.001	Lateral vs medial	< 0.001	1.366	1.174	1.590
		Combined vs medial	0.092	1.201	0.971	1.487
		Lateral vs combined	0.296	1.137	0.893	1.447
BMI [5-points-OR]	< 0.001			1.101	1.044	1.161
Drainage	0.073	Yes vs no		1.155	0.987	1.352
Gender	0.093	Female vs male		0.899	0.794	1.018
Risk factors	0.120	Yes vs no		1.108	0.974	1.261
Preoperative pain	0.238	Yes vs unknown	0.098	0.836	0.677	1.034
		Unknown vs no	0.261	1.135	0.910	1.416
		Yes vs no	0.444	0.949	0.831	1.084
ASA score	0.279	III/IV vs I	0.148	1.200	0.937	1.537
		III/IV vs II	0.202	1.096	0.952	1.261
		II vs I	0.416	1.096	0.879	1.365
Age [10-years-OR]	0.428			0.980	0.931	1.031

### Pain at rest

The analysis results for pain at rest at 1-year follow-up are summarized in Table 9 (model fit: *p* < 0.001). This was highly and significantly associated with age, preoperative pain, gender, EHS classification, postoperative complications, and ASA score (*p* < 0.001), as well as with BMI (*p* = 0.003), operative technique (*p* = 0.027), use of a drain (*p* = 0.032), and also hernia width (*p* = 0.033) Fig. 2). Higher age and higher BMI led to less pain at rest. On the other hand, preoperative pain, lateral EHS, as well as combined classification compared with medial, postoperative complications, and the use of a drain showed an increased risk of pain at rest. Besides, women were at higher risk of pain at rest at 1-year follow-up than men. The association between operative technique and risk of pain at rest was reflected primarily in the reduced risk posed by the open suture technique. Finally, width classification was also found to exert a significant influence on pain at rest, but that significant impact was identified only on comparing W3 vs. W1 and the impact was of a lesser degree, with 106 out of 1000 patients with W3 classification suffering from pain at rest compared to 90 out of 1000 patients with W1.

**Table 9:** Multivariable analysis results for pain at rest at 1-year follow-up, including odds ratio estimates with corresponding 95% confidence intervals

Parameter	*p* value	Category	*p* value (pairwise)	OR estimate	95% CI	
Age [10-years-OR]	< 0.001			0.799	0.771	0.827
Preoperative pain	< 0.001	Yes vs no	< 0.001	1.680	1.513	1.865
		Unknown vs no	< 0.001	1.413	1.186	1.685
		Yes vs unknown	0.036	1.189	1.012	1.397
Gender	< 0.001	Female vs male		1.516	1.384	1.660
EHS classification	< 0.001	Lateral vs medial	< 0.001	1.503	1.346	1.677
		Lateral vs combined	0.015	1.246	1.043	1.487
		Combined vs medial	0.020	1.206	1.030	1.412
Postoperative complication	< 0.001	Yes vs no		1.599	1.380	1.852
ASA score	< 0.001	III/IV vs I	< 0.001	1.522	1.276	1.816
		III/IV vs II	< 0.001	1.209	1.090	1.342
		II vs I	0.003	1.259	1.079	1.469
BMI [5-points-OR]	0.003			0.944	0.908	0.981
Procedure	0.027	Open—suture vs laparoscopic—IPOM	0.002	0.763	0.641	0.907
		Open—suture vs open—sublay	0.003	0.770	0.649	0.914
		Open—suture vs open—IPOM	0.020	0.797	0.659	0.965
		Open—suture vs open—Onlay	0.024	0.762	0.602	0.965
		Open—IPOM vs laparoscopic—IPOM	0.575	0.957	0.820	1.117
		Open—IPOM vs open—sublay	0.632	0.966	0.840	1.112
		Open—onlay vs open—IPOM	0.684	1.046	0.842	1.299
		Open—sublay vs laparoscopic—IPOM	0.882	0.990	0.866	1.131
		Open—onlay vs open—sublay	0.912	1.011	0.835	1.223
		Open—onlay vs laparoscopic—IPOM	0.995	1.001	0.809	1.237
Drainage	0.032	Yes vs no		1.136	1.011	1.276
EHS width classification	0.036	W3 (> 10 cm) vs W1 (< 4 cm)	0.010	1.201	1.044	1.382
		W3 (> 10 cm) vs W2 (≥ 4–10 cm)	0.057	1.128	0.997	1.277
		W2 (≥ 4–10 cm) vs W1 (< 4 cm)	0.255	1.065	0.956	1.187
Risk factors	0.365	Yes vs no		1.045	0.950	1.149

### Pain on exertion

Pain on exertion at 1-year follow-up, (model fit: *p* < 0.001), was highly significantly associated with age, gender, preoperative pain, EHS classification, postoperative complications, hernia width, operative technique, use of a drain (in each case *p* < 0.001), as well as BMI (*p* = 0.004) and presence of risk factors (*p* = 0.004) (Fig. 2 and Table 10). Higher age and higher BMI indicate a reduced risk of pain on exertion. On the other hand, women had a notably higher risk of pain in comparison with men. Preoperative pain, lateral EHS and combined vs. medial, postoperative complications, higher width classification, and presence of at least one risk factor likewise increased the risk of pain on exertion. The use of drains was also associated with a higher risk of pain on exertion. Likewise, the operative technique was found to have a significant impact. Here, too, that was reflected in particular in the advantages conferred by the open suture procedure, as shown by the above estimates. The difference in cases of pain on exertion is about 44 for every 1000 hernias with W3 (199/1000) compared to W1 width classification (155/1000).

**Table 10:** Multivariable analysis results for pain on exertion at 1-year follow-up, including odds ratio estimates with corresponding 95% confidence intervals

Parameter	p value	Category	*p* value (pairwise)	OR estimate	95% CI	
Age [10-years-OR]	< 0.001			0.760	0.739	0.781
Gender	< 0.001	Female vs male		1.590	1.481	1.706
Preoperative pain	< 0.001	Yes vs no	< 0.001	1.566	1.447	1.696
		Unknown vs no	< 0.001	1.378	1.204	1.576
		Yes vs unknown	0.045	1.137	1.003	1.289
EHS classification	< 0.001	Lateral vs medial	< 0.001	1.510	1.383	1.648
		Combined vs medial	< 0.001	1.304	1.154	1.473
		Lateral vs combined	0.038	1.158	1.008	1.330
Postoperative complications	< 0.001	Yes vs no		1.421	1.258	1.606
EHS width classification	< 0.001	W3 (> 10 cm) vs W1 (< 4 cm)	< 0.001	1.351	1.210	1.509
		W2 (≥ 4–10 cm) vs W1 (< 4 cm)	< 0.001	1.192	1.095	1.298
		W3 (> 10 cm) vs W2 (≥ 4–10 cm)	0.011	1.134	1.029	1.249
Procedure	< 0.001	Open—suture vs laparoscopic—IPOM	< 0.001	0.726	0.635	0.831
		Open—direkte naht vs open—sublay	0.003	0.818	0.716	0.935
		Open—suture vs open—IPOM	0.005	0.808	0.696	0.937
		Open—suture vs open—onlay	0.009	0.782	0.649	0.941
		Open—sublay vs laparoscopic—IPOM	0.025	0.888	0.800	0.985
		Open—IPOM vs laparoscopic—IPOM	0.083	0.899	0.797	1.014
		Open—Onlay vs Laparoscopic—IPOM	0.388	0.929	0.786	1.098
		Open—onlay vs open—sublay	0.555	1.046	0.900	1.217
		Open—onlay vs open—IPOM	0.706	1.033	0.871	1.225
		Open—IPOM vs open—sublay	0.822	1.013	0.907	1.130
Drainage	< 0.001	Yes vs no		1.189	1.086	1.303
BMI [5-points-OR]	0.004			0.956	0.928	0.986
Risk factors	0.004	Yes vs no		1.115	1.035	1.200
ASA score	0.103	III/IV vs I	0.037	1.154	1.009	1.320
		III/IV vs II	0.142	1.064	0.980	1.155
		II vs I	0.168	1.084	0.966	1.217

### Chronic pain requiring treatment

The analysis results for pain requiring treatment are presented in Table 11 (model fit: *p* < 0.001). Here, too, age, gender, preoperative pain, EHS classification, postoperative complications, ASA score, and presence of risk factors (in each case *p* < 0.001), as well as the use of a drain (*p* = 0.002), BMI (*p* = 0.017), and EHS width classification (*p* = 0.035) had a significant relation to the rate of chronic pain requiring treatment (Fig. 2). Likewise, women were at notably higher risk of pain than men. The risk declined with increasing age and higher BMI. Preoperative pain, lateral, or combined EHS classification in comparison with medial, postoperative complications, higher ASA score, presence of at least one risk factor, and the use of drains were once again associated with a higher proportion of cases with chronic pain requiring treatment. Finally, higher width classification implied a higher risk of pain requiring treatment, but its significant difference was identified only on comparing W3 vs. W1. This corresponds to 80 out of 1000 patients with W3 suffering from pain requiring treatment compared to 67 out of 1000 patients with W1 width.

**Table 11:** Multivariable analysis results for chronic pain requiring treatment at 1-year follow-up, including odds ratio estimates with corresponding 95% confidence intervals

Parameter	*p* value	Category	*p* value (pairwise)	OR estimate	95% CI	
Age [10-Jahres-OR]	< 0.001			0.787	0.756	0.819
Gender	< 0.001	Female vs male		1.741	1.568	1.932
Preoperative pain	< 0.001	Yes vs no	< 0.001	1.943	1.717	2.198
		Unknown vs no	< 0.001	1.529	1.246	1.877
		Yes vs unknown	0.011	1.271	1.056	1.529
EHS classification	< 0.001	Lateral vs medial	< 0.001	1.564	1.382	1.769
		Combined vs medial	< 0.001	1.376	1.159	1.635
		Lateral vs combined	0.198	1.136	0.936	1.379
Postoperative complication	< 0.001	Yes vs no		1.570	1.331	1.851
ASA score	< 0.001	III/IV vs I	< 0.001	1.804	1.465	2.221
		II vs I	< 0.001	1.448	1.204	1.741
		III/IV vs II	< 0.001	1.246	1.109	1.400
Risk factors	< 0.001	Yes vs no		1.220	1.097	1.356
Drainage	0.002	Yes vs no		1.230	1.079	1.403
BMI [5-points-OR]	0.017			0.950	0.910	0.991
EHS width classification	0.035	W3 (> 10 cm) vs W1 (< 4 cm)	0.012	1.228	1.047	1.439
		W2 (≥ 4–10 cm) vs W1 (< 4 cm)	0.067	1.122	0.992	1.270
		W3 (> 10 cm) vs W2 (≥ 4–10 cm)	0.207	1.094	0.951	1.258
Procedure	0.102	Open—suture vs open—IPOM	0.032	0.794	0.643	0.981
		Open—suture vs laparoscopic—IPOM	0.035	0.811	0.668	0.985
		Open—IPOM vs open—sublay	0.064	1.157	0.991	1.351
		Open—sublay vs laparoscopic—IPOM	0.107	0.883	0.759	1.027
		Open—suture vs open—Onlay	0.363	0.884	0.677	1.153
		Open—suture vs open—Sublay	0.389	0.919	0.759	1.113
		Open—onlay vs open—IPOM	0.390	0.899	0.705	1.146
		Open—onlay vs laparoscopic—IPOM	0.487	0.918	0.721	1.168
		Open—onlay vs open—sublay	0.724	1.040	0.837	1.293
		Open—IPOM vs laparoscopic—IPOM	0.808	1.021	0.860	1.213

### Summary of results

Figure 2 shows the main results from the aforementioned models in combination with the outcome of the bootstrap validation algorithm.

Higher EHS width classification (W2 vs. W1, W3 vs. W1) had a highly significantly unfavorable relation to the intraoperative and postoperative surgical complications, the complication-related reoperations, as well as to the general complications. On comparing W3 vs. W1, the former was additionally found to have a highly significantly unfavorable association with the complication-related reoperation rate, which in the case of W2 vs. W1 was only significantly unfavorable. Those model and bootstrap results thus demonstrate that EHS width classification has the highest negative association with the perioperative outcome in incisional hernia repair. Only the open vs. the laparoscopic technique likewise showed a highly significantly unfavorable relation to the postoperative surgical complication and the complication-related reoperation rates. Conversely, the open procedure compared with the laparoscopic technique had a highly significantly favorable association with the intraoperative complication rate. The only other significantly unfavorable relations identified were higher age to the intraoperative complications, higher ASA score to the general complications, risk factors to the postoperative surgical complications, complication-related reoperation rate, and the general complications. In contrast, lateral versus medial EHS classification had a significantly favorable association with the postoperative surgical complications, complication-related reoperation rate, and the general complications.

For the recurrence rate at 1-year follow-up, only higher EHS width classification, higher BMI, and lateral EHS classification were found to have a significantly unfavorable association, while the open vs. laparoscopic access technique showed a significantly favorable difference for the open approach.

The rates of pain on exertion, pain at rest, and chronic pain requiring treatment were highly significantly unfavorably associated with preoperative pain, female gender, and postoperative complications. Higher EHS width classification, risk factors, and lateral EHS classification had a significantly unfavorable association with the pain rates at 1-year follow-up. Higher age and higher BMI had a significantly favorable relation to pain rates.

### Subgroup of patients without follow-up

In order to investigate whether there are relevant differences between the analysis population (limited to those patients with follow-up information *n* = 22,895) and the subgroup of patients without 1-year follow-up (*n* = 13,637, Fig. 1), standardized differences were calculated for all patient- and operation-related variables, as well as post- and perioperative outcome variables. With the exception of age, with a mean difference of 2.4 years, for all other factors the standardized difference was found to be below 0.1. Thus, there is no bias in selection of patients due to the availability of follow-up information. The slightly higher age in the subgroup without follow-up demonstrates more difficulties in obtaining information related to outcome from older patients.

## Discussion

The present multivariable analysis of 22,895 primary elective incisional hernia repairs from the Herniamed Registry investigated the potential influencing factors associated with outcome.

Multivariable models were estimated based on the confirmatory chosen, potential influencing parameters using all available data according to inclusion/exclusion criteria. The robustness of the results was assessed using a bootstrap algorithm per model with 1000 bootstrap samples each.

Higher EHS defect width classifications were found to have a highly significantly unfavorable relation to intraoperative complications, postoperative surgical complications as well as to general complications. Furthermore, defect width > 10 cm had a highly significantly unfavorable association with the complication-related reoperation rate. Defects of ≥ 4–10 cm were found to have only a significantly unfavorable relation to the complication-related reoperation rate. Only the open vs. the laparoscopic procedure was found to have, additionally, a highly significant unfavorable association with the postoperative surgical complications and the complication-related reoperation rate. Conversely, the open technique had a highly significantly favorable association with the intraoperative complication rate. Hence, the EHS width classification and surgical access route were identified as being the most important influencing factors for the perioperative outcome in incisional hernia repair. Higher patient age, the presence of risk factors, and higher BMI can also unfavorably influence the perioperative outcome.

In this analysis, no highly significant influencing factor was identified for recurrence at 1-year follow-up. Here, too, a larger defect width, higher BMI, and lateral EHS classification were seen to be significantly associated with recurrence.

The most important influencing factors for pain at rest, pain on exertion, and chronic pain requiring treatment at 1-year follow-up were preoperative pain and female gender. Other significantly unfavorable influencing factors were higher EHS width classification, presence of risk factors, higher ASA score, and lateral EHS classification.

As such, EHS width classification, open surgical technique, patient-reported preoperative pain, and female gender are the most important influencing factors for the outcome in incisional hernia repair. Accordingly, the findings presented here can be put to use for risk adjustment in incisional hernia repair. However that presupposes preoperative determination of the defect size by means of ultrasonography, computed tomography, or magnetic resonance imaging [22–26]. Based on the radiologically measured defect width, the EHS width classification can then be used to estimate the expected outcomes.

Thus, this also serves as a good basis for the physician–patient consultation with regard to modification of risk factors, such as smoking and obesity, prior to surgery [13, 14]. In particular, in the case of large defects, other risk factors should be reduced as far as possible [27]. Since incisional hernias become larger over time [28], with correspondingly poorer outcomes, watchful waiting should be carefully considered [29].

Based on the present analysis, patients with an incisional hernia who are at higher risk for perioperative complications and an unfavorable outcome at 1-year follow-up can be identified. Such patients should be operated on by experienced hernia surgeons. In particular, that applies to incisional hernia patients with a defect width of > 10 cm and who according to the guidelines [30, 31] should be operated on with an open technique. These patients have the highest perioperative complication risk, but are also susceptible to a significantly unfavorable influence on the recurrence rate and pain rates at 1-year follow-up. Independently of the defect width, female patients and patients with reported preoperative pain are at higher risk for the onset of chronic pain requiring treatment.

Missing or incorrect data limit a registry [20]. All responsible surgeons participating in the Herniamed Registry sign a contract for data correctness and completeness [20]. Missing data are indicated by the registry software [20]. Postoperative complications are once again reviewed at 1-year follow-up [20]. Experts can control data entry as part of the certification process of hernia centers [20]. The lack of follow-up in a relevant percentage (Fig. 1) of patients is another limitation of this registry study, but the subgroup analysis does not show any selection bias. The best safeguard is to compare the data with the literature [20]. The findings presented here are in concordance with the published data [12–15].

Furthermore, registry analyses do not allow for causal inference, but associations of variables can at least be detected when adjusting for known confounders and can thus be discussed.

In conclusion, this analysis of data from the Herniamed Registry demonstrates the very unfavorable association between high EHS width classification and intraoperative, postoperative and general complications, and complication-related reoperations, as well as its unfavorable relation to recurrences and pain rates at 1-year follow-up. Pain at rest and on exertion, as well as chronic pain requiring treatment is very unfavorably associated with female gender and preoperative pain and unfavorably related to lateral EHS classification and high ASA score. In comparison with the laparoscopic approach, the open sublay technique showed a highly, significantly reduced intraoperative complication rate, but highly increased postoperative complication and complication-related reoperation rates. Because incisional hernias become larger over time [28], with correspondingly poorer outcomes, watchful waiting should be carefully considered in incisional hernia repair. Patients with highly significant unfavorable factors should be treated by an experienced hernia surgeon.
